# An Investigation of Levetiracetam in Alzheimer’s Disease (ILiAD): a double-blind, placebo-controlled, randomised crossover proof of concept study

**DOI:** 10.1186/s13063-021-05404-4

**Published:** 2021-07-31

**Authors:** Arjune Sen, Mary Akinola, Xin You Tai, Mkael Symmonds, Gabriel Davis Jones, Sergio Mura, Joanne Galloway, Angela Hallam, Jane Y. C. Chan, Ivan Koychev, Chris Butler, John Geddes, Rohan Van Der Putt, Sian Thompson, Sanjay G. Manohar, Eleni Frangou, Sharon Love, Rupert McShane, Masud Husain

**Affiliations:** 1 Oxford Epilepsy Research Group, NIHR Biomedical Research Centre, John Radcliffe Hospital, Oxford, UK; 2 Department of Neurology, John Radcliffe Hospital, Oxford, OX3 9DU UK; 3 Nuffield Department of Clinical Neuroscience, University of Oxford, Oxford, OX3 9DU UK; 4 Local Clinical Trials Network, John Radcliffe Hospital, Oxford, OX3 9DU UK; 5 Department of Clinical Neurophysiology, John Radcliffe Hospital, Oxford, OX3 9DU UK; 6 Clinical Trials Pharmacy, John Radcliffe Hospital, Oxford University Hospitals NHS Foundation Trust, Oxford, OX3 9DU UK; 7 CPSU, Oxford Health Foundation Trust, Oxford, OX1 5RW UK; 8 St Mary’s Pharmaceutical Unit, Cardiff University, Cardiff, 20 Fieldway, Cardiff, CF14 4HY UK; 9 Freeline Therapeutics, King’s Court, London Road, Stevenage, SG1 2NG UK; 10 Translational Medicine, UCB Pharma, 208 Bath Road, Slough, SL1 3WE UK; 11 Department of Psychiatry, University of Oxford, Oxford, OX3 7JX UK; 12 Faculty of Medicine, Department of Brain Sciences, Imperial College, Sir Alexander Fleming Building, South Kensington Campus, London, SW7 2BU UK; 13 Department of Psychiatry, University of Oxford, Warneford Hospital, Oxford, OX3 7JX UK; 14 Memory and Cognition Research Delivery Team, Warneford Hospital, Warneford Lane, Headington, Oxford, OX3 7JX UK; 15 MRC Clinical Trials Unit at UCL, Institute of Clinical Trials & Methodology, Faculty of Pop Health Sciences, University College London, London, UK; 16 Centre for Statistics in Medicine, Nuffield Department of Orthopaedics, Rheumatology and Musculoskeletal Sciences, University of Oxford, Windmill Road, Oxford, OX3 7LD UK; 17 Cognitive Neurology Research Group, Nuffield Dept Clinical Neurosciences & Department of Experimental Psychology, University of Oxford, West Wing, John Radcliffe Hospital, Oxford, OX3 9DU UK

**Keywords:** Anti-seizure medication, Cognition, Dementia, Epilepsy, Levetiracetam, Randomised controlled trial, Pilot, Proof of concept, Seizure

## Abstract

**Background:**

Although Alzheimer’s disease affects around 800,000 people in the UK and costs almost £23 billion per year, currently licenced treatments only offer modest benefit at best. Seizures, which are more common in patients with Alzheimer’s disease than age matched controls, may contribute to the loss of nerve cells and abnormal brain discharges can disrupt cognition. This aberrant electrical activity may therefore present potentially important drug targets. The anti-seizure medication levetiracetam can reduce abnormal cortical discharges and reverse memory deficits in a mouse model of Alzheimer’s disease. Levetiracetam has also been shown to improve memory difficulties in patients with mild cognitive impairment, a precursor to Alzheimer’s disease. Clinical use of levetiracetam is well-established in treatment of epilepsy and extensive safety data are available. Levetiracetam thus has the potential to provide safe and efficacious treatment to help with memory difficulties in Alzheimer’s disease.

**Methods:**

The proposed project is a proof of concept study to test whether levetiracetam can help cognitive function in people with dementia. We plan to recruit thirty patients with mild to moderate Alzheimer’s disease with no history of previous seizures or other significant co-morbidity. Participants will be allocated to a double-blind placebo-controlled crossover trial that tests levetiracetam against placebo. Standardised scales to assess cognition and a computer-based touchscreen test that we have developed to better detect subtle improvements in hippocampal function will be used to measure changes in memory. All participants will have an electroencephalogram (EEG) at baseline. The primary outcome measure is a change in the computer-based touchscreen cognitive task while secondary outcomes include the effect of levetiracetam on mood, quality of life and modelling of the EEG, including time series measures and feature-based analysis to see whether the effect of levetiracetam can be predicted. The effect of levetiracetam and placebo will be compared within a given patient using the paired t-test and the analysis of covariance adjusting for baseline values.

**Discussion:**

This is the first study to evaluate if an anti-seizure medication can offer meaningful benefit to patients with Alzheimer’s disease. If this study demonstrates at least stabilisation of memory function and/or good tolerability, the next step will be to rapidly progress to a larger study to establish whether levetiracetam may be a useful and cost-effective treatment for patients with Alzheimer’s disease.

**Trial registration:**

ClinicalTrials.govNCT03489044. Registered on April 5, 2018.

## Administrative information

The order of the items has been modified to group similar items (see http://www.equator-network.org/reporting-guidelines/spirit-2727-statement-defining-standard-protocol-items-for-clinical-trials/).

**Table:** 

Title {1}	An Investigation of Levetiracetam in Alzheimer’s Disease (ILiAD: a double-blind, placebo-controlled, randomised crossover proof of concept study
Trial Registration {2a and 2b}	ClinicalTrials.gov Identifier: NCT03489044; Registered: 5th April 2018. EudraCT Number: 2016-003109-32
Protocol version {3}	Version 5; 17.3.2020
Funding {4}	Medical Research Council, United Kingdom; UCB Pharma provided levetiracetam and placebo; Wellcome Trust
Author details {5a}	Dr Mary Akinola, General Practitioner with Special Interest in Dementia, Local Clinical Trials Network, John Radcliffe Hospital, Oxford. OX3 9DU Dr Xin You Tai, Neurology Specialist Registrar, Department of Neurology, John Radcliffe Hospital, Oxford. OX3 9DU Dr Mkael Symmonds, Consultant Neurologist and Neurophysiologist, Department of Clinical Neurophysiology, John Radcliffe Hospital, Oxford. OX3 9DU Dr Gabriel Jones, Research Scientist, Nuffield Department of Clinical Neuroscience, University of Oxford, Oxford. OX3 9DU Mr Sergio Mura, Clinical Trials Specialist Pharmacist, John Radcliffe Hospital, Oxford University Hospitals NHS Foundation Trust, Oxford. OX3 9DU Ms Joanne Galloway, Clinical Trials Pharmacist, CPSU, Oxford Health Foundation Trust, Oxford. OX1 5RW. Dr Angela Hallam, Head of Production, St Mary’s Pharmaceutical Unit, Cardiff University, Cardiff, 20 Fieldway, Cardiff. CF14 4HY Dr Jane Y C Chan, Medical Director, Freeline Therapeutics, King’s Court, London Road, Stevenage. SG1 2NG Previously: Medical Director, Translational Medicine, UCB, 208 Bath Road, Slough SL1 3WE Dr Ivan Koychev, Consultant Psychiatrist, Department of Psychological Medicine, John Radcliffe Hospital, Oxford. OX3 9DU Dr Chris Butler, Clinical Senior Lecturer in Dementia Research, Faculty of Medicine, Department of Brain Sciences, Imperial College, Sir Alexander Fleming Building South Kensington Campus, London. SW7 2BU Professor John Geddes, Head of Department, Department of Psychiatry, University of Oxford, Warneford Hospital, Oxford, OX3 7JX Dr Rohan Van Der Putt, Consultant Psychiatrist, Memory and Cognition Research Delivery Team, Warneford Hospital, Warneford Lane, Headington. Oxford. OX3 7JX Dr Sian Thompson, Consultant Neurologist, Department of Neurology, John Radcliffe Hospital, Oxford. OX3 9DU Dr Sanjay Manohar, Consultant Neurologist, Department of Neurology, John Radcliffe Hospital, Oxford. OX3 9DU Miss Eleni Frangou, Research Fellow (Medical Statistician, Clinical Trials), MRC Clinical Trials Unit at UCL, Institute of Clinical Trials &Methodology, Faculty of Pop Health Sciences, University College London, London and Centre for Statistics in Medicine, Nuffield Department of Orthopaedics, Rheumatology and Musculoskeletal Sciences, University of Oxford, Windmill Road, Oxford, OX3 7LD Dr Sharon Love, Senior Statistician, Centre for Statistics in Medicine, Nuffield Department of Orthopaedics, Rheumatology and Musculoskeletal Sciences, University of Oxford, Windmill Road, Oxford, OX3 7LD, Professor Rupert McShane, Consultant Psychiatrist, Department of Psychiatry, University of Oxford, Warneford Hospital, Oxford, OX3 7JX Professor Masud Husain, Consultant Neurologist, Cognitive Neurology Research Group, Nuffield Dept Clinical Neurosciences & Department of Experimental Psychology, University of Oxford, New Radcliffe House, 1st Floor Radcliffe Observatory Quarter, Oxford, OX2 6GG
Name and contact information for the trial sponsor {5b}	Clinical Trials & Research Governance, University of Oxford, Joint Research Office, Boundary Brook House, Churchill Drive, Headington. Oxford. OX3 7GB
Role of the sponsor {5c}	Sponsor ensured concordance with good clinical practice and monitoring of study. The funders had no role in study design or data capture

## Introduction

### Background and rationale {6a}

Alzheimer’s disease (AD) is the most common neurodegenerative disease [1]. The socioeconomic impact is vast. Many patients live for 10 years after diagnosis and the cost per annum for each patient with AD is more than the median UK salary [1]. However, the only currently licenced treatments for AD offer modest benefit at best. Given the paucity of effect of currently available treatments, finding a novel therapeutic agent for AD represents one of the most pressing of current global health issues.

As well as memory loss, seizures are more frequent in patients with Alzheimer’s disease. In the developed world, the incidence of seizures is highest in the older population [2]. However, seizures are three times more common in patients with AD aged 80 than those without dementia and the absolute risk of seizures in people with AD is approximately 10 to 20% [3]. Patients with Alzheimer’s disease may also have evidence of abnormal cortical activity detectable with an electroencephalogram (EEG) without having obvious clinical seizures [reviewed in 2]. Similarly, neuronal hyperactivity in the hippocampus and medial temporal lobe structures might be an important precursor to cognitive decline in AD [reviewed in 4].

Seizures and seizure-like EEG activity were considered a consequence of the loss of nerve cells in the brain of a patient with Alzheimer’s disease. However, seizures may contribute directly to the synaptic reorganisation, network disruption, neuronal loss, and cognitive deficits observed in AD [4]. Excitotoxicity, as is often observed in seizure-mediated neuronal loss, can cause tau tangle formation—a molecular marker of AD [5]. Molecular cascades important in AD are also deregulated in epilepsy [6–8]. In older patients with focal cortical dysplasia (a common cause of drug-resistant epilepsy), tau tangles and neuronal loss are observed within the dysplastic cortex, but not in adjacent histologically normal tissue, further suggesting a convergence of molecular processes important in epileptogenesis and neurodegeneration [7, 9].

All anti-seizure medications aim to achieve therapeutic function by stabilising neuronal networks. Older medications, for example phenobarbitone, had significant adverse effect profiles and could impair cognitive function. Modern anti-seizure medications are much better tolerated and most do not tend to adversely affect cognition, even in older patients [reviewed in 2]. The potent, established anti-seizure medication levetiracetam binds synaptic vesicle protein 2A (SV2A) and inhibits N-type calcium channels to impede synaptic transmission. Intriguingly, SV2A can contribute causally to late-onset AD by mediating the impact of apolipoprotein E4 on amyloid precursor protein (APP) [10].

Initial trials of levetiracetam in epilepsy recruited more than 1000 patients with significant reduction in seizures compared to placebo (Table 1). The drug was well-tolerated with main adverse events being somnolence and fatigue. Subgroup analyses showed that levetiracetam may be particularly efficacious in those aged over 65 years [14]. Levetiracetam has no adverse impact on cognition (including in older subjects) [17]. In individuals with AD and seizures, levetiracetam associated with improvements in attention and verbal fluency as well as better seizure control [18]. It is, however, difficult to determine whether levetiracetam offered additional cognitive benefits beyond that associated with seizure control alone. Also, levetiracetam has no known pharmacokinetic interactions and clinical use over more than a decade has confirmed it to be effective and well-tolerated. Levetiracetam, which is now available as a generic medication, is widely prescribed and there is considerable familiarity with the medication from primary through to tertiary care.

**Table 1:** Summary of most relevant clinical trials using levetiracetam (LEV) demonstrating both efficacies and tolerability in epilepsy

Authors and year	Total no. of patients/ no. given LEV	Dose (mg/day)	Impact of LEV on seizure frequency	Significant adverse events
**Ben-Menachem and Falter, 2000** [11]	286/181	3000	Significant benefit of LEV vs. placebo both as add-on (p < 0.001) and as subsequent monotherapy (p = 0.037)	Incidence of adverse events similar in placebo and treatment groups
**Betts et al., 2000** [12]	119/80	2000 or 4000	Significant benefit of LEV vs. placebo at 2000 mg per day (p < 0.05)	Somnolence, asthenia
**Cereghino et al., 2000** [13]	294/199	1000 or 3000	Significant benefit of LEV vs. placebo (50% responder rate p < 0.001)	Somnolence, asthenia, infection e.g. rhinitis
**Ferrendelli et al., 2003** [14]	78 /78 (patients older than 65 years)	1000 to 3000	Subset analysis of patients who participated in the open label KEEPER trial of a total of 1030 patients.50% responder rate of 76.9%	Somnolence, asthenia Medication well-tolerated in older patients
**Shorvon et al., 2000** [15]	324/212	1000 or 2000	Significant benefit of LEV vs. placebo	No difference in adverse effects vs. placebo. Main side effects: somnolence, asthenia
**Cochrane review (meta-analysis 11 trials incl. above), 2012** [16]	1861 (1565 adults)	1000–4000	Significant benefit to seizures from LEV at every dose compared to placebo Improved cognitive outcomes in adults	Somnolence (RR 1.51; 99% CI 1.06 to 2.17) Infection (RR 1.76; 99% CI 1.03 to 3.02)

Owing to its lack of drug-drug interactions and favourable cognitive profile, levetiracetam is a preferred medication in older people with epilepsy [14]. However, the interest in utilising levetiracetam in AD is founded on animal experiments. Levetiracetam decreases amyloid plaques and alleviates behavioural deficits in an APP-transgenic model of AD [19]. Levetiracetam has also been shown to reduce abnormal electrographic activity and reverse learning and memory deficits in the human APP-transgenic mouse [20]. Such effects were not observed with other anti-seizure medications that were tested [20].

Clinical evaluations of levetiracetam in AD have been limited to people with overt clinical seizures. Levetiracetam is effective at controlling seizures and is well-tolerated in people with epilepsy and AD [18, 21, 22]. Importantly, levetiracetam can improve memory function in people with mild cognitive impairment at risk of developing AD potentially through changes in hippocampal activation [23].

We therefore propose performing a double-blind proof of concept crossover study to evaluate whether levetiracetam may be beneficial to cognition in patients with mild to moderate AD who do not have epilepsy or a history of seizures. To shorten trial duration, refined measures of cognition will be employed to detect any subtle differences in patients while taking levetiracetam compared to placebo.

We hypothesise that:

1. Treatment with the anti-seizure medication levetiracetam will be of benefit to cognitive deficits in Alzheimer’s disease (AD) because electrophysiological disruption of neuronal networks contributes to the pathophysiology of the condition.
2. Treatment with levetiracetam at a dose that is routinely utilised in older people with epilepsy will be well-tolerated in patients with AD and, in particular, will not adversely affect mood
3. Electroencephalography (EEG) may offer biomarkers to predict which patients with AD might be especially suited to treatment with levetiracetam

## Methods: participants, interventions and outcomes

### Objectives {7}

#### Primary

1. To determine if the anti-seizure medication levetiracetam offers benefit to cognition in patients with AD who have not experienced an overt seizure

#### Secondary

1. To evaluate if use of levetiracetam associates with significant side effects in patients with AD that have not experienced an overt seizure
2. To determine if use of levetiracetam associates with an effect on mood in patients with AD that have not experienced an overt seizure
3. To determine if use of levetiracetam associates with changes in quality of life in patients with AD that have not experienced an overt seizure, or a change in the quality of life of their carers
4. To evaluate whether EEG can be used as a surrogate marker to better predict which patients with AD may respond to treatment with levetiracetam

### Trial design {8}

ILiAD is a randomised double-blind placebo-controlled crossover study to evaluate whether treatment with levetiracetam is superior to placebo in its effect on memory function in people with AD. The study is counterbalanced and participants either receive placebo or levetiracetam first. Data are compared, for a given individual, from when the participant is taking levetiracetam to when taking placebo. Given the overall short duration of the study, it is not anticipated that there will be marked progression of the dementia during the study.

### Study setting {9}

This pilot study is conducted only in Oxford at two hospital sites—Oxford University Hospitals NHS Foundation Trust and Oxford Health NHS Foundation Trust. Where possible site visits can be held at home.

### Eligibility criteria {10}

#### Inclusion criteria

All participants:

- Participant is willing and able to give informed consent for participation in the trial.
- Participant speaks English as their first language

Participants with AD

- Male or female, 50 years or above.
- Diagnosed with mild to moderate AD (Mini-Mental State Examination score of 10 to 26)
- Meets the National Institute of Aging-Alzheimer’s Association criteria for probable AD (2011)
- Stable dose of current regular medication, including acetylcholinesterase inhibitors if applicable, for at least 4 weeks prior to trial entry.
- Female participants of child bearing potential and male participants whose partner is of child bearing potential must be willing to ensure that they or their partner use effective contraception during the trial and for 3 months thereafter.
- Participant has clinically acceptable blood and urine test results (creatinine clearance > 75 ml/min; liver function tests < 2× upper limit of normal) and ECG that does not demonstrate conduction block or significant ischaemia within 3 months of enrolment.
- In the Investigator’s opinion is able and willing to comply with all trial requirements.
- Willing to allow his or her General Practitioner and consultant, if appropriate, to be notified of participation in the trial.
- Reliable carer willing and available to assist with medication administration as well as to accompany participants during any home visits.

Carer of participant with AD

- Male or female aged 18 and above.
- Principal carer for the participant with AD
- Able to attend all home visits

#### Exclusion criteria

The participant may not enter the trial if ANY of the following apply.

Participants with AD

- Pre-existing diagnosis of epilepsy
- Clinical or laboratory evidence of a cause other than AD as a cause of their dementia
- Laboratory evidence of significant renal impairment (creatinine clearance < 75 ml/minute) or liver dysfunction (liver function tests > 2× upper limit of normal) within the preceding 3 months
- Visual or motor impairment that investigator deems severe enough to impair ability to complete computerised based touchscreen task
- Use of anti-seizure medication for any indication (epilepsy, pain or migraine) within the previous 3 months
- Other severe neurological or medical condition. Examples include significant stroke, heart failure, chronic renal failure, chronic liver failure within last 3 months
- Major depression or other significant behavioural disturbance
- Known allergy to levetiracetam or history of previous adverse reaction to levetiracetam
- Female participant who is pregnant, lactating or planning pregnancy during the course of the trial
- Scheduled elective surgery or other procedures requiring general anaesthesia during the trial.
- Participant with life expectancy of less than 6 months, or is inappropriate for placebo medication.
- Any other significant disease or disorder which, in the opinion of the Investigator, may either put the participants at risk because of participation in the trial, or may influence the result of the trial, or the participant’s ability to participate in the trial
- Participants who have participated in another research trial involving an investigational medicinal product in the past 12 weeks

Carer of participant with AD

- Carer has significant medical illness that will preclude adequate data capture during the study

### Who will take informed consent? {26a}

Medical personnel who have completed all of the relevant training to understand the protocols and safety requirements of the ILiAD study will obtain written informed consent. Only people who can provide informed consent will be recruited. All personnel involved in the project will also have completed up-to-date Good Clinical Practice and relevant trials training. Separate information sheets and consent forms will be provided for AD patients and carers.

### Additional consent provisions for collection and use of participant data and biological specimens {26b}

Participants will consent to sharing anonymised data with collaborators and colleagues and will be given the option to be contacted about future studies should they so wish.

## Interventions

### Explanation for the choice of comparators {6b}

We would not expect to see major changes in Alzheimer’s Disease Assessment Scale–Cognitive Subscale (ADAS-Cog, a standard measure of cognition applied in AD research) over 24 weeks. Therefore, more sensitive markers of cognition will be employed including a neuropsychological test measuring binding of features which has been shown to be important in memory associated with hippocampal function [24], including in familial AD [25]. We have accounted for a possible attrition of 20% and a cohort of 30 participants is adequately powered to detect a meaningful difference of 0.7 in the hippocampus-dependent memory-binding test. Standardised and widely deployed scales to measure quality of life and psychological/psychosocial factors will also be utilised

### Intervention description {11a}

The selected dose of levetiracetam is informed by well-established clinical practice. A dose of levetiracetam at 500 mg twice daily is generally considered the lowest therapeutic dose for people with overt seizures and is well-tolerated by elderly individuals with epilepsy [14]. Therefore, it is reasonable to hypothesise that levetiracetam at 500 mg twice daily will modulate potentially epileptogenic networks. Studies in patients with AD and epilepsy have generally had a mean levetiracetam dose of 1000 mg daily (Table 1), and in these studies, this dose has again been well-tolerated.

Trials of levetiracetam in patients with MCI are beginning with dosing of levetiracetam 250 mg twice per day. We hypothesise that in AD the neuronal networks are less stable than in MCI and hence a higher dose of levetiracetam should initially be tested in patients with AD. The dose proposed of levetiracetam 500 mg twice daily is therefore realistic in terms of titration schedule and tolerability and offers the best opportunity to determine if levetiracetam can change cognition through stabilisation of neuronal networks in patients with AD.

**Fig. 1:**
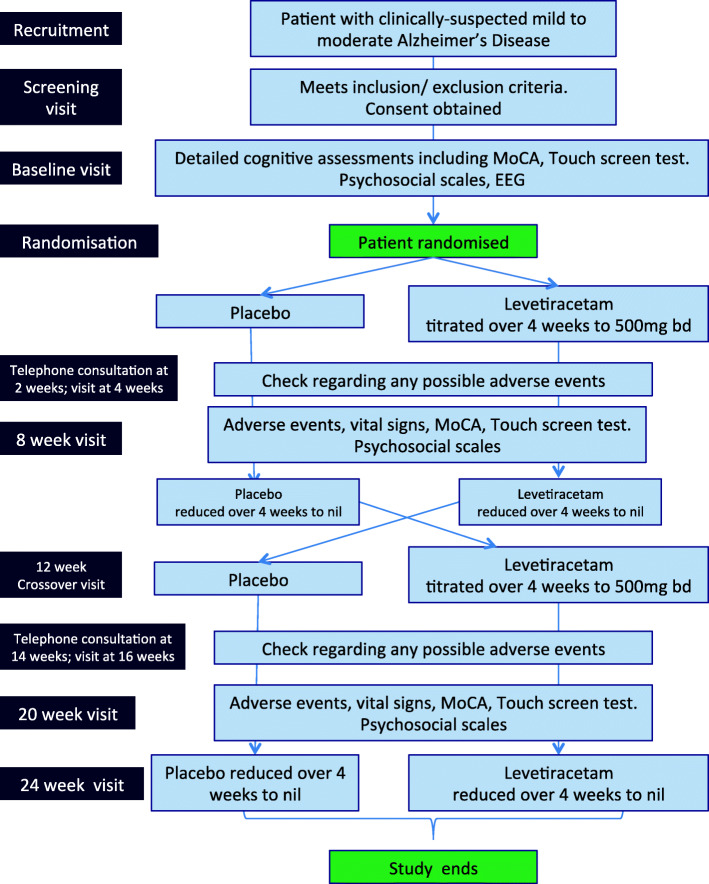
ILiAD trial outline. The ILiAD trial is a double-blind, placebo-controlled crossover study with study visits each month. It is a designed to provide pilot data to determine whether there may be a role for levetiracetam, an anti-seizure medication, in helping memory problems in people with Alzheimer’s disease (EEG = electroencephalogram; MoCA = Montreal Cognitive Assessment)

**Fig. 2:**
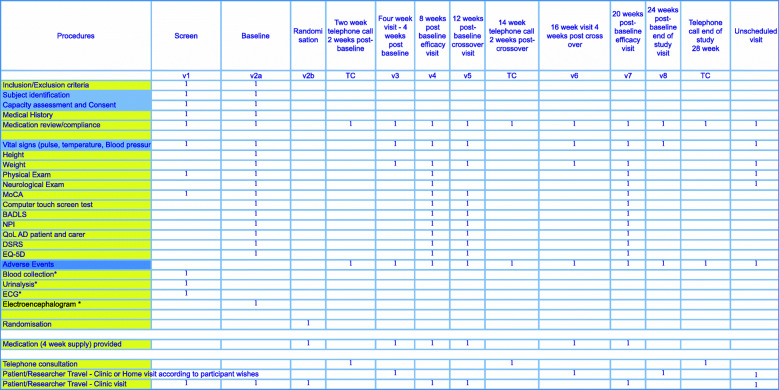
ILiAD visit schedule The tasks to be completed by the participant, carer and researcher are listed. Visits can typically be completed within 2 h although the screening and baseline visit can take around 4 h. IMP is provided at each visit also. * = tests will not be repeated if acceptable results are already available from within 3 months of recruitment date. Abbreviations: TC = telephone consultation; BADLS = Bristol Activities of Daily Living Scale; DSRS = Dementia Severity Rating Scale; MoCA = Montreal Cognitive Assessment; NPI = Neuropsychiatry Inventory; QoL = Quality of Life; EQ-5D = Euro-QoL Quality of Life Measure

A trial flow chart and a schedule of trial procedures and visit schedule are provided in Figs. 1 and 2 respectively.

We aim to recruit 30 patients (15 in each arm) to a randomised, double-blind placebo-controlled crossover study as illustrated. Study duration is 28 weeks and in total there are 11 scheduled contacts with investigators (8 face-to-face encounters, of which 3 may be at the participant’s home should they so wish, and 3 are telephone consultations). The time and date of all visits and assessments is recorded for all participants.

Measures taken at baseline will be as follows:

1. Computerised touchscreen test of memory binding [24]. This computer-based test, which is available to the ILiAD Consortium as it was developed by one of the PIs (MH), enables detection of subtle changes in hippocampal function and will help delineate whether levetiracetam has offered benefit
2. Montreal Cognitive Assessment (MoCA)
3. Bristol Activities of Daily Living Scale (BADLS)
4. Neuropsychiatric Inventory (NPI)
5. EEG recording (unless performed in the last 3 months)

Quality of life estimates for both the patient and the carer (if participating) will be undertaken using the Dementia Severity Rating Scale (DSRS) and Euro-Qol Quality of Life measure (EQ5D}. The timing of all interventions is as detailed on the flowsheets.

All participants will have a baseline EEG. The benefit of EEG in AD is not certain, but novel modelling approaches are gaining traction. By acquiring these data prior to commencing treatment/placebo, we will look to determine whether there are EEG biosignatures that predict responsiveness to levetiracetam.

Blood samples (10 ml for analysis of full blood count, urea, creatinine, electrolytes, liver function tests, blood glucose) and a standard urinanalysis (30 ml urine) will be collected once, at the screening visit. These samples are collected to ensure that there is no evidence of sub-clinical renal or hepatic impairment that may not have been apparent from review of the notes alone. Samples will be disposed of using the disposal facilities of the recruiting site as per standard clinical care. The blood and urine samples are only provided to confirm eligibility and will not be retained.

**Fig. 3:**
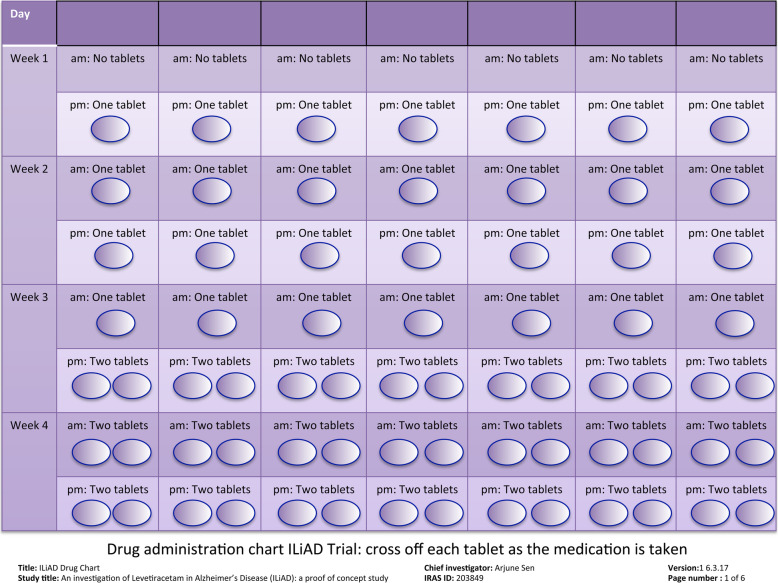
ILiAD drug administration chart with each provision of IMP, carers are provided with a drug administration chart. Each day, the carer crosses off the tablet on the chart as the IMP is given to the participant. Researchers review these charts at the end of each month to ensure concordance with IMP administration. The example above is for weeks 1 to 4 in the study

Randomisation will occur at or shortly after the baseline visit. Levetiracetam and placebo will be packaged with clear instructions to take tablets as outlined (Table 2; Fig. 3). Each tablet of levetiracetam is 250 mg. Participants will commence on one tablet at night and increase by one tablet every week to two tablets twice daily (Table 2; Fig. 3). They will then continue on two tablets twice daily for 4 weeks before reducing by one tablet each week until the IMP is withdrawn. The participant will then cross over to either levetiracetam or placebo, whichever they were not initially allocated to, and complete the same up- and down-titration schedule. Given the short half-life of levetiracetam (6 to 8 h (SMPC)) and the assessment schedule, which includes repeat testing prior to entry into the second arm of the trial, a washout period after the first arm was not thought necessary.

**Table 2:** Planned titration regime of IMP during ILiAD study

	Daily morning dose (each tablet 250 mg)	Daily evening dose (each tablet 250 mg)
**Week 1**	Nil	One tablet
**Week 2**	One tablet	One tablet
**Week 3**	One tablet	Two tablets
**Week 4**	Two tablets	Two tablets
**Week 5**	Two tablets	Two tablets
**Week 6**	Two tablets	Two tablets
**Week 7**	Two tablets	Two tablets
**Week 8**	Two tablets	Two tablets
**Week 9**	One tablet	Two tablets
**Week 10**	One tablet	One tablet
**Week 11**	Nil	One tablet
**Week 12**	Nil	Nil

### Subsequent visits

There will be safety checks at week 2, week 4, week 14, and week 16. These timepoints coincide with patients receiving levetiracetam 250 mg twice daily or 500 mg twice daily. Should they so wish, some of the visits may be at the patient’s home or remotely by telephone. If any visits are at the patient’s home, then the carer must also be present throughout that visit. If the visit is conducted remotely by telephone, the researcher should complete all assessments that are possible to complete by telephone as per the schedule of assessments.

At weeks 8 and 20, patients and carers will be required to attend the clinical research site for detailed assessments including all of the measures of efficacy performed at baseline as well as detailed reporting of potential side effects. In the event that visits cannot be conducted at the clinical research site, the visits can be conducted remotely by telephone. The visits should still be conducted as per the schedule of assessments and by staff delegated to complete the specified study tasks. Any assessments not completed need to be documented as a protocol deviation. At week 24, there will be a further telephone consultation with patients who have completed the trial.

In brief the visits can be summarised as follows:

1. Screening visit: patient screened against inclusion/exclusion criteria
2. Baseline and randomisation visit: eligible patients who consent to participation are evaluated as outlined and randomised to either receive levetiracetam first or placebo first. Baseline data are captured as detailed in baseline assessments
3. Telephone call 2 weeks after randomisation: as levetiracetam/placebo is uptitrating, there will be a telephone call at 2 weeks to ensure no adverse effects and document adherence
4. 4-week visit when participants in the active arm will have reached levetiracetam 500 mg twice daily.
5. Eight week efficacy visit to evaluate effect of levetiracetam/placebo using all of the measures performed at the baseline visit.
6. 12-week crossover visit. Participants who were randomised to levetiracetam will have weaned to nil and start to take placebo and vice versa. Vital signs, adverse effects and adherence will be recorded. The cognitive and neuropsychiatric measures undertaken at baseline will be repeated to ensure no ‘carry over’ effect from patients who received levetiracetam initially.
7. Telephone call 14 weeks after randomisation: as levetiracetam/placebo is uptitrating, there will be a telephone call at 2 weeks to ensure no adverse effects and document adherence
8. 16-week visit when participants in the active arm will have reached levetiracetam 500 mg twice daily.
9. 20-week efficacy visit (8 weeks after cross over visit) to evaluate effect of levetiracetam/placebo using all of the measures performed at the baseline visit.
10. 24-week end-of-study visit, 24 weeks post baseline. At this visit, patients will have weaned from either levetiracetam or placebo completely. Vital signs, medication adherence and adverse effects will be recorded.
11. Telephone call at 28 weeks after randomisation, 4 weeks after weaning from study medication to document any adverse events from medication

Participants can also make unscheduled visits if required. These visits will be triggered by the participant at any stage through the trial should concerns, particularly relating to possible adverse effects, arise. If an unscheduled visit is required, dependent on the reason for the visit, the visit may occur at the clinical research site, at the participant’s home or telephonically.

### Criteria for discontinuing or modifying allocated interventions {11b}

Each participant has the right to withdraw from the trial at any time. If a participant chooses to withdraw from the study, we will assume that the participant is taking the active drug. In addition, the Investigator may discontinue a participant from the trial at any time if the Investigator considers it necessary for any reason including:

- Pregnancy
- Ineligibility (either arising during the trial or retrospectively having not been apparent/declared at screening)
- Significant protocol deviation
- Significant non-adherence with treatment regimen or trial requirements
- An adverse event which requires discontinuation of the trial medication or results in inability to continue to comply with trial procedures
- Disease progression which requires discontinuation of the trial medication or results in inability to continue to comply with trial procedures
- Patient experiences an epileptic seizure necessitating the initiation of anti-seizure medication
- Withdrawal of consent
- Loss of capacity to consent to trial procedures—if the investigator determines that a participant has lost the capacity to consent, then the participant will exit the trial.
- Loss to follow-up

If treatment is withdrawn, the medication will reduce by one tablet per week until weaned away completely. Participants will be contacted 1 week after withdrawal of medication to ensure that there have been no significant adverse events.

If treatment is withdrawn and participants exit the trial, no further data will be captured. Data already acquired will be included in the analysis. Investigators will aim to recruit so that 30 participants complete the trial if possible. A minimum of 24 participants is required to complete the entire duration of the trial to ensure that analysis is meaningful. If the total number of participants remaining in the study falls below 24, additional participants will be recruited aiming to ensure that a minimum of 24 patients complete the study.

The reason for withdrawal, if given, will be recorded in the CRF. If the participant is withdrawn due to an adverse event, the Investigator will arrange for follow-up visits or telephone calls until the adverse event has resolved or stabilised.

### Strategies to improve adherence to interventions {11c}

To improve adherence to medication the carer of the patient will be given a tablet chart (Fig. 3) to help the accurate administration of medication. Tablets will be counted on return, which will further clarify concordance with the planned dosing. The involvement of the carer will also help ensure concordance with other aspects of the study.

### Relevant concomitant care permitted or prohibited during the trial {11d}

Levetiracetam does not interact pharmacokinetically with many other medications. Should acute treatments be needed, for example a course of antibiotics, the participant will continue in the trial. A file note will be made of the start date and end date of any such treatments. Participants must not be involved in other clinical trials while recruited to ILiAD.

### Definition of end of trial

The end of trial is the date of the last telephone follow-up of the last participant.

### Provisions for post-trial care {30}

The trial does not progress to an open label extension study. Were the trial to yield promising data, then a much larger study to thoroughly evaluate the efficacy of levetiracetam in this participant group will be performed. The University of Oxford has a specialist insurance policy in place which would operate in the event of any participant suffering harm as a result of their involvement in the research. NHS indemnity operates in respect of the clinical treatment that is provided.

### Outcomes {12}

#### Primary

1. Changes in cognition in patients while taking levetiracetam as measured by computerised assessment of hippocampal binding memory task. Measures will be compared as change from baseline in each arm of the study (namely compare end of arm 1 to baseline and end of arm 2 to measures taken at start of arm 2). Were the study to show that levetiracetam can stabilise or even improve cognitive testing on a very specific task that evaluates hippocampal function that would open an entirely new therapeutic avenue for people with Alzheimer’s disease.

#### Secondary

1. Determination of side effects from levetiracetam in the study population compared to placebo. Anti-seizure medications are generally well-tolerated in people with epilepsy, but are not routinely administered to people who have not had a seizure. It is therefore clinically essential to determine if there are side effects in this patient group. This will be enquired about at each study contact.
2. Determination of the effect on mood of levetiracetam in the study population compared to placebo using standardised scales such as Neuropsychiatry Index. Given that levetiracetam can have an adverse effect on mood, this will be specifically evaluated and monitored throughout. Principal comparisons will be between the start and end of each arm of the study.
3. Determination of the effect on quality of life from levetiracetam in the study population compared to placebo using standardised scales such as the BADLS, EQ-5D and DSRS index (compare end of arm 1 to baseline and end of arm 2 to end of arm 1). Even were levetiracetam to not show an advantage on cognitive measures, were it to improve quality of life in people with AD that may make it a worthwhile treatment.
4. Assessment and modelling of the EEG prior to starting levetiracetam and correlation with response to levetiracetam. It may well be that levetiracetam is not suitable for all people with AD. EEG could offer a simple non-invasive biomarker to better determine who may benefit from levetiracetam and similarly who may experience side effects. The EEG is performed at baseline and will be modelled and correlated with outcomes for each participant at study end.

### Participant timeline {13}

The participant timeline and visit schedules are illustrated in Figs. 1 and 2.

### Sample size {14}

The trial is relatively short and one would not anticipate being able to detect changes in ADAS-Cog over this time-frame. Therefore, the trial is powered to detect a standardised effect of 0.7 in a specific and sensitive test of hippocampal function that has already been tested in people with AD [24, 25]. The estimated variability of 2% was taken from data on a memory-binding task for older individuals (aged 60–83) [26]. The standard deviation of the difference between the treatment and placebo was then calculated assuming equal variability in the two groups. This yielded an estimate of 3%. Using the two-sided test for paired means in PASS 11, the total sample required to detect a mean difference of 2.1% with a standard deviation of the difference of 3% and achieve 90% power at a 5% significance level in 24 patients.

Therefore, a sample size of 30 allows an attrition rate of 20% for the study still to be able to detect a meaningful difference in sensitive measures of cognitive function.

### Recruitment {15}

Participants will be recruited from Oxford University Hospitals NHS Foundation Trust (OUHFT) and Oxford Health NHS Foundation Trust (OHFT). The same method of recruitment will be applied at both sites.

Potentially eligible patients will be approached by a clinician who knows them and will be provided with an opportunity to hear more about the study. The initial approach will usually be made at the routine clinic visit for that patient. Eligible participants may initially be approached without their carer being present as all recruited patients must have capacity to consent to the ILIAD trial at screening.

Those interested will be provided with the PIS and either meet with a member of the research team on the same day (if the participant is able to do so) or given instructions on how to contact a member of the research team and arrange to meet at a mutually convenient time. The researcher will then go through the PIS and provide further information about the study. This meeting will occur in the out-patient clinic setting.

If the patient wishes to participate, they will be asked to provide verbal consent for the researcher to review their medical notes and screen against basic inclusion/exclusion criteria. If the patient appears eligible, they will be asked to give informed consent be enrolled into the trial. The patient will then undergo full screening including necessary blood tests, ECG recording and urinalysis. Should any patients, on full screening, be found to not meet all eligibility criteria, those patients will then be withdrawn.

At the same time as the patient is recruited, the patient’s carer will also be invited to participate in appropriate aspects of the study.

## Assignment of interventions: allocation

### Sequence generation {16a}

As ILiAD is a crossover trial, patients will be randomised 1:1 levetiracetam: placebo. All tablets of levetiracetam and placebo will appear identical. Restricted block randomisation will be performed by the Centre for Statistics in Medicine, University of Oxford, who will generate a series of randomisation codes. The investigators and patients will be blinded to treatment allocation.

### Concealment mechanism {16b}

**Fig. 4:**
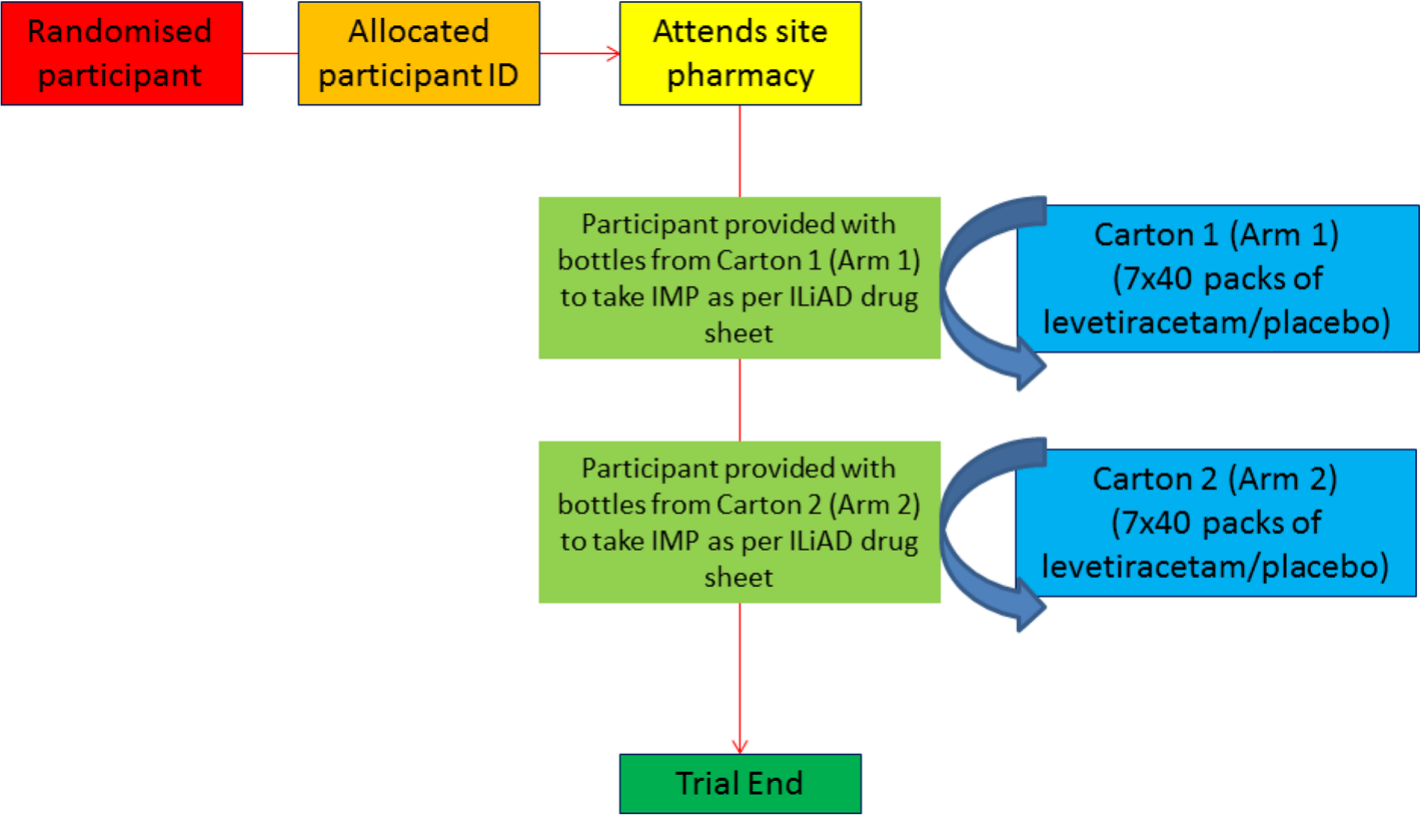
Administration of IMP during the ILiAD trial. Randomised participants will attend site pharmacies to collect IMP as outlined. Each participant has a designated carton held at the trial pharmacies. IMP is randomly allocated as placebo or levetiracetam to arm1 and to arm 2. Participants then collect IMP from carton 1 and then carton 2 and titrate medication/placebo as per the administration schedules

The generated codes will be given to the Clinical Trial Pharmacist at St Mary’s Pharmaceutical Unit, Cardiff and Vale University Health Board, who will allocate each code to a pair of cartons of medication. Each of these two cartons will be labelled with the same randomisation code. One of the two cartons will be labelled Arm 1 and will contain seven packs of medication with each pack containing 40 tablets of levetiracetam/placebo. The other of the two cartons will be labelled Arm 2 and will contain seven packs of medication with each pack containing 40 tablets of levetiracetam/placebo. Within a given pair of cartons, if the carton labelled Arm 1 contains levetiracetam then the carton labelled Arm 2 will contain placebo and vice versa. This is illustrated in Fig. 4.

### Implementation {16c}

The allocation system will be generated by the Centre for Statistics in Medicine, University of Oxford. The research team will enrol participants and recruited participants will be given a sequential identification number. Randomisation will occur after the baseline visit. The PI enrolling the patient will complete the ILiAD randomisation form. The site Pharmacy will dispense sequentially according to the Participant Trial ID Number.

## Assignment of interventions: blinding

### Who will be blinded? {17a}

Trial participants, care providers, principal investigators and all attending staff will be blinded to treatment allocation as none will know the code underpinning the treatment allocation. Investigators, patients and carers will remain blinded to the treatment allocation throughout the trial.

### Procedure for unblinding if needed {17b}

Unblinding should not normally be necessary as serious side effects will be dealt with on the assumption that the patient is on active treatment. Study medication should be omitted rather than unblinded. Request for unblinding should be directed to the Study Office during office hours. Each recruiting site will hold trial information for that site.

Given that levetiracetam is a well-established anti-seizure medication with a known side effect profile and is therefore considered a relatively low-risk IMP, out of hours code breaking is not thought to be required. However, to mitigate against any possible risk to participants, the main site, at the John Radcliffe Hospital, will have the CI’s mobile telephone number held at their Switchboard to enable prompt discussion about potential unblinding.

If a patient wishes to be unblinded for non-clinical reasons, the PI for that patient will submit a written request to the CI who will evaluate each participant wishing to be unblinded on a case by case basis.

Unblinding of a single patient will not lead to unblinding for the whole trial.

### Data collection and management

#### Plans for assessment and collection of outcomes {18a}

All trial staff will receive training in how to conduct the scales and assessments at a site initiation visit. The questionnaires to be administered in ILiAD (Addenbrooke’s Cognitive Estimate [27]; Bristol Activity of Daily Living Scale [28]; Dementia Severity Rating Scale [29]; Neuropsychiatric Inventory [30]; EQ5D [31]; Quality of life scale in AD [32]) are already used widely in clinical practice, and the computer-based task has been validated as a measure of hippocampal function [24–26].

Assessments will be completed according to the visit schedule.

#### Plans to promote participant retention and complete follow-up {18b}

The ILiAD study involves close co-operation between the participant and the research team. Potential participants and their carers will be fully informed of the visit schedule which we hope will ensure good retention of those recruited.

All randomised participants will be included in the analysis. Patients who withdraw from the study at any stage will be asked for permission for their data accumulated to the point of withdrawal to be included. Data from patients who have been unblinded will be included to the point of unblinding.

## Data collection and management {19}

Source documents include, but are not limited to, hospital records (from which medical history and previous and concurrent medication may be summarised into the CRF), clinical and office charts, laboratory and pharmacy records, diaries, microfiches, radiographs and correspondence. CRF entries will be considered source data if the CRF is the site of the original recording (e.g. there is no other written or electronic record of data). All documents will be stored safely in confidential conditions. On all trial-specific documents, other than the signed consent, the participant will be referred to by the trial participant number/code, not by name.

Each participant will have a designated trial folder. Carers will be allocated the same identification number as the participant followed by the word ‘CARER’. Data for cognitive, neuropsychiatric and psychosocial measures will be entered onto paper CRFs. These paper CRFs will be stored in the individual participant’s folder. Blood test results (with normal reference ranges) performed at screening will be printed onto a single sheet and stored in the participant’s folder. A redacted ECG performed within 3 months of enrolment will also be placed on file.

All documents will be stored securely and information from paper CRFs will be entered into the online RedCap electronic CRF system which is accessed securely through the University of Oxford webserver. Data entry into RedCap will be additionally supervised by the Trial Manager to ensure that there are no transcription errors. The participants will be identified only by a participant ID number on the paper CRF and any electronic database. Participants’ identification numbers and personal identification details will be stored separately in encrypted files on a password protected Oxford University server.

### Confidentiality {27}

The trial staff will ensure that the participants’ anonymity is maintained. The participants will be identified only by a participant ID number on all trial documents and any electronic database. Carers will have the same participant ID number as the participant with AD, followed by the word ‘CARER’. All documents will be stored securely and only accessible by trial staff and authorised personnel. The trial will comply with the Data Protection Act, which requires data to be anonymised as soon as it is practical to do so.

All data captured in the ILiAD study will be kept for 5 years after the end of the study whereupon it will be destroyed according to University of Oxford standard disposal procedures

### Plans for collection, laboratory evaluation and storage of biological specimens for genetic or molecular analysis in this trial/future use {33}

The only laboratory tests performed are blood tests at screening. The samples will be analysed by standard methods in clinical laboratories. Samples will be considered a gift to the study and destroyed after analysis according to standard NHS disposal methods. There is therefore no planned storage of biological materials for use in the current trial or in future studies.

## Statistical methods

### Statistical methods for primary and secondary outcomes {20a}

A trial-specific statistician based at the Centre for Statistics in Medicine, University of Oxford, will perform statistical analysis.

As the trial is crossover in design, all analyses will be within participants, comparing outcome measures after taking the placebo and after taking levetiracetam. Thus, the analyses of the primary and secondary outcomes relating to cognition, behaviour and quality of life will be based on the differences between the observations at the efficacy visits (week 8 and week 20) of the levetiracetam and placebo periods.

Within a given patient, levetiracetam and placebo will be compared using the paired t-test and the analysis of covariance adjusting for baseline values. We will compare results at 8 weeks to the initial baseline testing and at 20 weeks to the further baseline testing at 12 weeks. All results will be presented with 95% confidence intervals. For each carer who has consented to provide data, quality of life measures will likewise be compared between the observations at the week 8 and week 20 visits. Appropriate sensitivity analyses will be performed to confirm the robustness of the data.

EEG will be computationally analysed, after standard cleaning, downsampling and artefact rejection. Typical EEG data features of interest include time series measures (for example, mean, variance, skewness, kurtosis and coastline), frequency based features (e.g. band-passed power and rhythmicity) and channel pair-wise bivariate connectivity metrics. These baseline EEG features will be correlated with clinical outcome measures.

### Interim analyses {21b}

No interim or additional analyses are planned as this is a small pilot study.

### Methods for additional analyses (e.g. subgroup analyses) {20b}

Subgroup analysis will be performed according to the EEG findings of participants and may also be performed for certain specific clinical parameters for example duration of disease.

### Methods in analysis to handle protocol non-adherence and any statistical methods to handle missing data {20c}

Missing data will be sought and supplemented where possible after consultation with the investigator. The control of the correctness of the data will be performed by central monitoring procedures. Unused data will be retained in the same way that used data is retained.

### Plans to give access to the full protocol, participant level-data and statistical code {31c}

On completion of the study, the entire protocol will be provided as an open access appendix to any arising manuscripts. Irretrievably anonymised datasets analysed during the current study are available from the corresponding author on reasonable request.

## Oversight and monitoring

### Composition of the coordinating centre and trial steering committee {5d}

The lead site is the John Radcliffe Hospital, Oxford University Hospitals NHS Foundation Trust. An annual report will be submitted to the Monitor who will also have access to all study data on request. The Oxford University Hospitals Trust / University of Oxford Trials Safety Group (TSG) will conduct a review of all SAEs for the trial reported during the quarter and cumulatively. The aims of this committee include:

- To detect any trends, such as increases in un/expected events, and take appropriate action
- To seek additional advice or information from investigators where required
- To evaluate the risk of the trial continuing and take appropriate action where necessary

### Composition of the data monitoring committee (DMC), its role and reporting structure {21a}

Given the short nature of the trial, the small number of planned participants and that the IMP is widely prescribed in clinical practice, it was agreed that a DMC was not required.

### Adverse event reporting and harms {22}

Levetiracetam, as outlined, is a well-tolerated and widely prescribed anti-seizure medication and is a drug of choice in older people with epilepsy. The safety and tolerability profile of levetiracetam is therefore very well-established and familiar to both investigators and General Practitioners. Common side effects from levetiracetam include asthenia, an effect on sleep and an effect on mood. Gastrointestinal disturbance, change in weight and rash are occasionally reported. The effect of levetiracetam on mood in patients with AD is captured within the data that will be collected from recruited participants. The full list of potential expected adverse events is listed in the Summary of Product Characteristics [33].

Given the familiarity with levetiracetam in both tertiary and primary care and, in particular, given that levetiracetam is being used at its licenced dose, non-serious adverse events will not be recorded within the ILiAD study unless they lead to withdrawal from treatment. Solicited and unsolicited adverse events are listed in Table 3 below:

**Table 3:** Solicited and unsolicited potential adverse events associated with levetiracetam

Adverse event	Solicited	Unsolicited
**Asthenia**		x
**Change in weight**		x
**Cough**		x
**Effect on mood/behaviour**	x	
**Effect on sleep**		x
**Gastrointestinal disturbance**		x
**Nasopharyngitis**		x
**Rash**	x	

SAEs will be recorded from the time of taking informed consent to 28 days following the last administration of study medication. The participants in this study are from an older population and may have co-morbidities beyond Alzheimer’s disease. An event that is part of the natural course of the disease (i.e. disease progression or admission for standard treatment) will not be reported as an SAE. However, if the progression of the underlying disease is greater than that which would normally be expected, or if the investigator considers that there may be a causal relationship between the IMP or protocol design/procedures and the disease progression, then it must be reported.

Reportable SAEs will be reported on the SAE reporting form to the trial monitor within 24 h of the Site Study Team becoming aware of the event. The trial monitor will perform an initial check of the report, request any additional information, and ensure it is reviewed by the Medical Monitor on a weekly basis. It will also be reviewed at the next Trial Safety Group meeting. All SAE information must be recorded on an SAE form and emailed to CTRG. Additional and further requested information (follow-up or corrections to the original case) will be detailed on a new SAE Report Form and faxed/emailed to CTRG.

All SUSARs will be reported by the CI to the relevant Competent Authority and to the REC and other parties as applicable. For fatal and life-threatening SUSARS, this will be done no later than 7 calendar days after the Sponsor or delegate is first aware of the reaction. Any additional relevant information will be reported within 8 calendar days of the initial report. All other SUSARs will be reported within 15 calendar days. Treatment codes will be unblinded for specific participants.

Principal investigators will be informed of all SUSARs for the relevant IMP for all studies with the same Sponsor, whether or not the event occurred in the current trial.

### Frequency and plans for auditing trial conduct {23}

Owing to the short nature of the trial, audits of trial conduct are not planned. The trial will subject itself to any necessary monitoring or audit deemed necessary and appropriate by the Oxford Trials Safety Group.

### Plans for communicating important protocol amendments to relevant parties (e.g. trial participants, ethical committees) {25}

Protocol amendments will, once approved by the sponsor, be sent to the Health Research Authority and the Research Ethics Committee that initially approved the protocol (Oxford REC B; Oxford, UK). Approved amendments will be communicated to all investigators and study researchers, the trial monitor and, where there is a material change in participant activities or requirements, to the participants and their carers.

Similarly, were there to be a change necessitating informing the relevant regulators (MHRA), then appropriate documentation would be completed through the European CESP portal and actioned. Information on clinicaltrials.gov, which is in the public domain, will be maintained and updated as required.

### Dissemination plans {31a}

Results will be presented in manuscripts, abstracts, press releases and other media with particular assurance that material is accessible to non-specialists. Authorship will be determined in accordance with the ICMJE guidelines and other contributors will be acknowledged

## Discussion

It has long been recognised that seizures are more common in older people with dementia [2, 3, 34]. More recently, it is appreciated that there is likely a bi-directional relationship between epilepsy and dementia [2, 34] and that both represent large-scale disruption of neuronal networks [34]. As such it seems tractable that anti-seizure medications, which help stabilise neuronal networks in epilepsy may offer some benefit to memory difficulties in people with dementia.

Basic science work suggests that levetiracetam, which works by binding to the protein synaptic vesicle protein 2A [35], holds the most promise as such a treatment in AD. Further benefits of levetiracetam include its limited side effect profile, ease of titration and lack of drug-drug interaction—this being particularly important in older people who may be taking multiple other medications and have significant co-morbidities. Levetiracetam is now available in generic preparations thus reducing the associated costs to healthcare providers. At a time when many are exploring very expensive immunological treatments for AD, it may be that the simple and inexpensive levetiracetam will at least offer some help to people with AD. To explore this, we developed the ILiAD trial.

ILiAD is very different to most studies in AD. It is short, involves repurposing of an existing widely prescribed medication and, as a crossover study, all participants have the opportunity to be given levetiracetam. While AD generally progresses slowly, the hippocampal binding task we employ in this study offers a very precise assessment of memory function [24] and this test can detect changes that may be apparent over a few months. The test may therefore be helpful in other AD studies going forwards. Similarly, EEG may provide a very helpful non-invasive biomarker to determine who may benefit from specific treatments in AD. Future, longer duration studies will also help evaluate the possible enduring impact of levetiracetam on cognition in people with AD as well as monitoring for any adverse events. We appreciate that by choosing to preferentially analyse data from participants who complete the study, there might be a bias towards people who tolerate levetiracetam. To mitigate this we will record all adverse events and the reasons why participants may have had to withdraw from the study before completion. As this is, very much, a pilot study all such data will inform future work exploring the potential role of levetiracetam in those with dementia.

Perhaps most importantly, irrespective of results, ILiAD opens an entirely new frontier in the treatment of Alzheimer’s disease bringing together the fields of epileptology and cognitive neurology. Were an anti-seizure medication, not necessarily levetiracetam, shown to positively influence memory in AD, this would be revolutionary. Moreover, as the utility of anti-seizure medications is in neuronal network stabilisation, independent of the cause of perturbation to that neuronal network, it may be that anti-seizure medications will also prove beneficial in other dementing illnesses beyond AD.

## Trial status

The ILiAD trial is currently open but not actively recruiting. The current protocol version number is 5. The first patient was recruited in December 2019. We anticipate completing recruitment in July 2022.

### Addendum: Adjustments owing to COVID-19 pandemic

At the time of initial submission, April 2020, owing to the coronavirus (COVID-19) pandemic, all ‘visits’ are now being performed telephonically to minimise any exposure of participants to hospital environments and to ensure that we do not burden NHS staff or facilities. IMP is delivered to the participant’s residence and there is no face-to-face contact between research personnel and participants. We are suspending all new recruitment to the study at the current time.

While we appreciate that these measures have a very significant impact on the trial, and if prolonged restrictions continue we may only be able to report safety data, we are nonetheless pleased that the study is being allowed to continue. Participants and their carers have invested considerably in the project and it would have been difficult had the trial stopped abruptly. Moreover, it remains important to complete research in populations who may be more vulnerable to COVID-19, but similarly are also more likely to have other health conditions that need addressment.

At the time of submitting the revised manuscript, ILiAD is planning on re-opening July 2021 with recruitment planned to end in July 2022. This is, though, contingent on how the COVID pandemic evolves over the next 18 months.

## Abbreviations

AD: Alzheimer’s disease
AE: Adverse event
AR: Adverse reaction
BADLS: Bristol Activities of Daily Living Scale
CI: Chief investigator
CRA: Clinical Research Associate (Monitor)
CRF: Case Report Form
CRO: Contract Research Organisation
CT: Clinical Trials
CTA: Clinical Trials Authorisation
CTRG: Clinical Trials and Research Governance
DMC/DMSC: Data Monitoring Committee / Data Monitoring and Safety Committee
DSRS: Dementia Severity Rating Scale
DSUR: Development Safety Update Report
EQ5D: Euro-Qol Quality of Life measure
GCP: Good Clinical Practice
GP: General practitioner
ICF: Informed consent form
IMP: Investigational Medicinal Product
MHRA: Medicines and Healthcare products Regulatory Agency
MOCA: Montreal Cognitive Assessment
NHS: National Health Service
NPI: Neuropsychiatric Inventory
NRES: National Research Ethics Service
PI: Principal investigator
PIS: Participant/ Patient Information Sheet
R&D: NHS Trust R&D Department
REC: Research Ethics Committee
SAE: Serious adverse event
SAR: Serious adverse reaction
SDV: Source Data Verification
SMPC: Summary of Medicinal Product Characteristics
SOP: Standard operating procedure
SUSAR: Suspected unexpected serious adverse reactions
TMF: Trial Master File
TSG: Oxford University Hospitals Trust / University of Oxford Trials Safety Group
